# Effects of propolis supplementation on prooxidant-antioxidant balance, oxidative stress biomarkers, and body composition in obese patients with NAFLD: A double-blind randomized controlled clinical trial

**DOI:** 10.34172/hpp.42549

**Published:** 2024-10-31

**Authors:** Hamideh Nazari-Bonab, Mahlagha Nikbaf-Shandiz, Helda Tutunchi, Mehrangiz Ebrahimi-Mameghani

**Affiliations:** ^1^Student Research Committee, Tabriz University of Medical Sciences, Tabriz, Iran; ^2^Endocrine Research Center, Tabriz University of Medical Sciences, Tabriz, Iran; ^3^Nutrition Research Center, Department of Biochemistry and Diet Therapy, Faculty of Nutrition and Food Sciences, Tabriz University of Medical Sciences, Tabriz, Iran

**Keywords:** Body composition, Liver function tests, Non-alcoholic fatty liver disease, Oxidative stress, Propolis

## Abstract

**Background:**

Oxidative stress is one of the main hits in the pathogenesis of non-alcoholic fatty liver disease (NAFLD). Propolis (PRP), a natural substance made by bees from plant extracts, has been reported to have antioxidant properties. The present clinical trial investigated the effect of Iranian PRP on prooxidant-antioxidant balance (PAB), oxidative stress biomarkers, and body composition in obese patients with NAFLD.

**Methods:**

In the present double-blind, randomized controlled clinical trial, 44 obese patients with NAFLD were randomly allocated to either Iranian PRP (1500 mg/d) or placebo (1500 mg/d) accompanied by a calorie-restricted diet (CRD) for eight weeks. PAB, oxidative stress biomarkers, and body composition were assessed at baseline and the end of the study.

**Results:**

There was a significant reduction in PAB levels over the trial in both groups. However, the between-group difference was not significant at the endpoint. At the end of the study, the inter-group comparison showed a significant decrease in serum glutathione peroxidase level in the placebo group compared to the PRP group after adjusting for confounding variables based on models 1 (*P*=0.027) and 2 (*P*=0.028). No significant within- or between-group differences in other studied oxidative stress biomarkers were found. Moreover, no between-group differences were observed for body composition and dietary intakes of energy and antioxidant micronutrients.

**Conclusion:**

Iranian PRP supplementation (1500 mg/d) for eight weeks could prevent the reduction of glutathione peroxidase levels compared to the control group. However, it could not affect other oxidative stress biomarkers, body composition, or dietary intakes of energy and antioxidant micronutrients.

## Introduction

 Non-alcoholic fatty liver disease (NAFLD) is defined as a set of conditions ranging from steatosis with no signs of liver cell damage to steatohepatitis with an unremitting inflammatory status to cirrhosis.^1-3^ The prevalence of NAFLD is estimated to range from 11.2% to 37.2% in the general population and 31% in the Asian population.^4^ It is the most common chronic liver disease among overweight people.^4,5^ Overweight and obesity, insulin resistance, dyslipidemia, inflammation, oxidative stress, arterial hypertension, and genetic factors affect not only the onset but also the progression of NAFLD.^1,2,5-8^

 Several studies have investigated the role of oxidative stress as an important factor in the pathology of metabolic conditions such as NAFLD.^9,10^ According to the “multiple-hit hypothesis”, lipid accumulation in hepatocytes leads to disruption of the mitochondrial antioxidant capacity as well as endoplasmic reticulum stress, exciting fat oxidation pathways.^11^ Oxidative stress causes an inflammatory response through the positive regulation of highly sensitive C-reactive protein (hs-CRP), proinflammatory cytokines such as interleukin-6 (IL-6), and tumor necrosis factor α (TNF-α).^12,13^

 Although there is no approved treatment for NAFLD, present management is based on lifestyle modification, including healthy dietary patterns and physical activity.^14^ Additionally, it appears that substances with antioxidant properties could be considered an effective way to control these pathways. Nowadays, there is a great concern about the possible role of natural compounds in preventing liver diseases.^15-17^ Propolis (PRP) is a natural viscous substance made by bees from plant extracts, flowers, and gums. The combination of PRP with bees’ saliva^18-21^ has been shown to contain different compositions depending on the plant source and geographical area.^22-25^ PRP is generally rich in flavonoids, phenolic acids, and terpenes, as well as proteins, sugars, minerals, and vitamins.^23,26^ A number of properties have been attributed to PRP, e.g., cytotoxicity, antioxidant, free radical scavenging properties, anti-inflammatory, immune stimulant, anti-tumor, liver protection, local anesthetic, antimicrobial, and antiviral activities.^18,27-29^ The medical benefits of PRP have encouraged studying the chemical compounds and their widespread clinical use in humans.

 Various components, particularly flavonoids and polyphenols, are responsible for its antioxidant and biological properties.^28^ These biological polyphenols exert their effects by chelating ionic metals, preventing the formation of free radicals, and inhibiting the enzymes involved in the initiation of free radical reactions.^30,31^ Various animal and in vitro studies indicate the positive effect of PRP on improving oxidative stress.^32-37^ Kismet et al observed that PRP significantly improved malondialdehyde (MDA) and glutathione peroxidase (GPX) levels in rats with NAFLD. Moreover, recent human studies have reported controversial findings regarding the effect of PRP with different dosages and various clinical conditions. Some have illustrated the protective effects of PRP on oxidative stress.^38-42^ For example, Afsharpour et al reported that PRP administration at 1500 mg in patients with type 2 diabetes mellitus (T2DM) for two months resulted in an improved antioxidant status.^38^ Likewise, Hesami et al observed a significant improvement in antioxidant status.^43^ However, Gao et al and Zhao et al did not observe significant results using 900 mg PRP for 16 weeks in patients with T2DM.^44,45^

 Although most studies have investigated antioxidant biomarkers, the prooxidant-antioxidant balance (PAB) method has been introduced. This method is a simple, fast, and inexpensive way to determine the oxidants and antioxidants simultaneously in one single test.^46^ Only one study by Darvishi et al reported that 8-week supplementation with PRP (500 mg/d) did not affect PAB in patients with breast cancer after chemotherapy.^47^

 The increasing trend in NAFLD prevalence worldwide has limited human studies on the effects of PRP supplementation on oxidative stress assessed by PAB compared with other biomarkers of oxidative stress, particularly in patients with NAFLD, and controversies in findings. The purpose of this study was to examine the effect of PRP supplementation on PAB, oxidative stress, body composition, and liver function in NAFLD patients.

## Material and Methods

###  Setting of the study

 This double-blind, placebo-controlled, randomized clinical trial is part of a previously published study.^48^ The target population was obese patients (n = 44) with NAFLD referred to sub-specialized and specialized clinics of Tabriz University of Medical Sciences from February 2021 to October 2021. Eligible subjects were patients with mild or moderate NAFLD diagnosed with ultrasound, aged 20-50, and a body mass index (BMI) between 30 and 40 kg/m^2^. NAFLD was diagnosed by a radiologist using the ultrasonography findings of Hamaguchi et al.^49^ All the patients were given a comprehensive explanation of the study objectives, and written informed consent was obtained from them. The study protocol was approved by the Ethics Committee of the Vice-Chancellor (Ethics code: TBZMED. REC. 1399.942). The protocol was also registered at the Iranian Registry of Clinical Trials (IRCT20100209003320N20).

 The exclusion criteria were as follows: suffering other chronic and acute liver diseases such as alcoholic fatty liver, Wilson’s disease, autoimmune liver disease, hepatitis, following a weight loss diet or supplements over the last three months, or taking hepatotoxic, lipid- or glucose-lowering medications and antioxidant supplements in the past three months. Additionally, individuals who were pregnant, lactating, athletes, postmenopause, cigarette smokers, alcohol drinkers, and allergic to PRP or honey products were also excluded.

###  Sample size

 Using power analysis and sample size software (PASS; NCSS, LLC, US), the sample size was estimated at 18 for each group, taking into account the mean change in GPX reported by Afsharpour et al,^38^ a 95% confidence level, and 90% power. Then, considering a 30% dropout rate, the sample size was increased to 23.

###  Intervention, randomization, and concealment

 An independent assessor assigned patients randomly into one of the two study groups,“PRP” or “Placebo,” in a 1:1 ratio using Random Allocation Software (RAS), in blocks stratified based on BMI (30–34.9 kg/m^2^*vs.* 35–39.9 kg/m^2^), age (20–35 years *vs.* 36–50 years), and gender (male *vs.* female). Both patients and investigators were blind to the group allocation until the end of the study. The person who was not involved in the study procedure and determined the eligibility and entry of patients, was responsible for allocation concealment, i.e. the assignments were enclosed in serially numbered, opaque, sealed envelopes with a 3-digit code to each of the treatments.

 The patients in the PRP group (n = 23) took 500 mg Iranian PRP capsules (containing 170 mg of poplar-type PRP ethanol extract and 330 mg oat and bee pollen), while those in the placebo group (n = 21) took 500 mg placebo capsules (containing cornstarch and food coloring) three times a day after each meal for 8 weeks. PRP and placebo supplements were similar in size, shape, and color, with a specified code. The dose and duration of the intervention were determined based on the previous study,^38^ and no side effects were observed with this dose. The adherence was consuming more than 90% of the supplements assessed by counting the capsules returned by the patients. Demographic details and an International Physical Activity Questionnaire (IPAQ) were completed at the beginning and end of the study.

 As a result, each participant received a personalized calorie-restricted diet (CRD) based on their resting metabolic rate, calculated using the Mifflin formula, physical activity level, and thermic effect of food (TEE) as 10% of total energy expenditure. To achieve weight loss, the estimated TEE was decreased to -500 kcal. The distribution of macronutrients in the designed CRD was 50% carbohydrates, 30% fat, and 20% protein. After preparing meal plans according to the food-based dietary guidelines for Iranians, a full explanation was given to each patient on how to use food exchange lists and how to replace the foods they did not have access to. A 3-day food record (2 weekdays and one weekend) was obtained at baseline and post-intervention to assess patients’ adherence to the prescribed diets. Dietary intakes were analyzed using Nutritionist IV software (First Databank, San Bruno, CA, USA) modified for Iranian foods. In addition, the patients were monitored for supplement consumption every 2 weeks during the intervention period.

###  Body composition and BMI

 BMI was calculated based on weight (kg) divided by the square of height (m^2^). Moreover, body composition was assessed using a bioelectric impedance analyzer (Japan, Takara BC-418) by asking patients to remove metal objects and place the soles of their feet and the palms of their hands directly on the electrodes of the device. Also, in order to prevent the device’s accuracy from being disturbed, the patients were required not to exercise for 90-120 minutes before the visit and to drink enough water.

###  Assessment of dietary intake and physical activity 

 A 3-day food record (two non-consecutive days of the week and one weekend) was completed at baseline and after 8 weeks for all patients to estimate energy, macronutrients, and dietary antioxidants.

 The short form of the International Valid Physical Activity Questionnaire (IPAQ-SF)^50^ was used to determine physical activity based on metabolic equivalent work minutes per week (MET-min/week).

###  Study outcomes

 The primary outcomes of this trial were changes in oxidative stress biomarkers and PAB levels. The secondary outcomes were changes in body composition, dietary energy intake, and antioxidant micronutrients.

###  Laboratory assays

 A blood sample was taken after 12-14 hours of fasting at the beginning and end of the study. The blood samples were put into tubes with or without EDTA. The EDTA-containing tubes were centrifuged at 3000 rpm for 10 minutes to separate serum, while whole blood samples were applied to determine GPX and superoxide dismutase (SOD) levels after obtaining hemoglobin concentration. Both whole blood and serum samples were stored at -80 °C until analysis. The activity of Cu/Zn-SOD and GPX was quantified using Ransod (Randox Laboratories, Ltd., UK) and Ransel commercial kits (Randox Laboratories, Ltd., UK, BT29 4QY), respectively. MDA concentration was assessed based on reaction with thiobarbituric acid^51^ using spectrophotometry, whereas serum levels of total antioxidant capacity (TAC) were determined using the Randox TAS kit (Randox Laboratories, Ltd., UK) and spectrophotometry. Serum levels of gamma glutamine transferase (GGT), alanine aminotransferase (ALT), and aspartate aminotransferase (AST) were assessed using the enzymatic method. Moreover, the NAFLD fibrosis score was estimated based on the following formula according to Angulo and colleagues’ study and classified into “low probability of advanced fibrosis” (≤ -1.455), “moderate probability of advanced fibrosis” (−1.455 to 0.676), and “high probability of advanced fibrosis” (≥ 0.676).^52^

 NAFLD fibrosis score = -1.675 + 0.037 × age (years) + 0.094 × BMI (Kg/m2) + 1.13 × impaired fasting glucose (IFG)/diabetes (yes = 1, no = 0) + 0.99 × AST/ALT ratio – 0.013 × platelet × (109/L) – 0.66 × albumin (g/dL).

 The serum PAB level was determined using the ELISA technique, following the method introduced and developed by Hamidi-Alamdari and colleagues.^46^

###  Statistical analyses 

 For statistical analysis, the SPSS IBM Statistics version 22 (Armonk, NY: IBM Corp) software and per-protocol principle were used. The Kolmogorov-Smirnov test was used to check the distribution of quantitative data. The data with a normal distribution was presented as mean ± SD, while asymmetric data was reported as median and interquartile range. To compare the variables between the two groups at the beginning of the study, the independent samples t-test and Mann-Whitney U test were applied, while intra-group changes were compared using paired sample t-test and Wilcoxon signed rank test. At the end of the study, ANCOVA to assess inter-group differences after adjusting for confounding variables based on two models: (1) adjusted for baseline values, and (2) adjusted for baseline values as well as energy intake.

## Results

**Figure 1:**
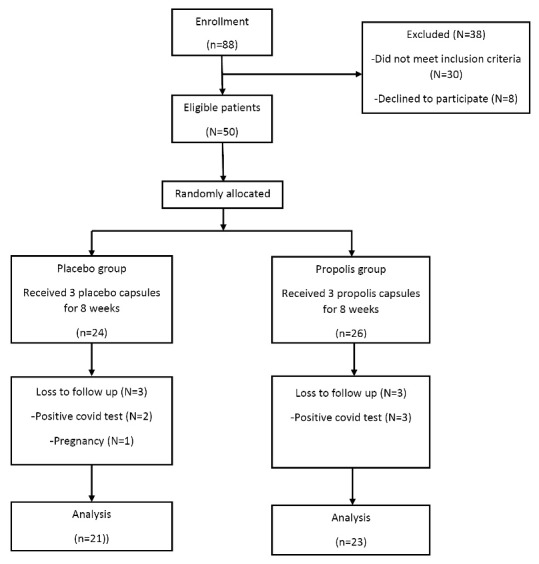
Trial profile and design

 Out of the 50 patients who enrolled in the study, three patients in the PRP group (due to COVID-19 infection) and two patients in the placebo group (due to COVID-19 infection and pregnancy) were excluded. Therefore, 44 patients completed the intervention (Figure 1). No side effects or symptoms were reported by the patients following PRP supplementation.

 As shown in Table 1, there were no statistically significant differences between the two groups in terms of body weight, BMI, age, sex, disease severity (in terms of NAFLD grade), fibrosis status, and physical activity level at baseline.

**Table 1:** Baseline characteristics of the study participants

**Variables**	**Propolis ** **(N=23)**	**Placebo** **(N=21)**	***P***
	**N (%)**	**N (%)**	
Gender, Female	15 (65.2)	13 (61.9)	0.821^b^
Education			
High school	11 (47.8)	13 (61.9)	0.349^b^
University degree	12 (52.2)	8 (38.1)	
Physical activity			
Low	17 (73.9)	17 (81.0)	0.724^c^
Moderate	6 (26.1)	4 (19.0)	
NAFLD severity			
Grade1	7 (30.4)	9 (42.9)	0.392^b^
Grade2	16 (69.6)	12 (57.1)	
Fibrosis status			
Low	19 (82.6)	17 (81.0)	1.000^c^
Moderate	4 (17.4)	4 (19.0)	
	**Mean ± SD**	**Mean ± SD**	
Age (years)	38.52 ± 7.51	40.14 ± 9.19	0.524^a^
Body weight (kg)	93.32 ± 15.27	89.02 ± 13.22	0.327^a^
BMI (kg/m^2^)	33.37 ± 4.82	33.00 ± 4.18	0.792^a^

BMI, body mass index. ^a^Independent *t *test. ^b^Chi-square. ^c^Fisher’s exact test.

 There were no significant inter-group differences in dietary intakes of energy, antioxidant micronutrients, or physical activity levels after 8 weeks of intervention (Table 2).

**Table 2:** Dietary intakes of energy, antioxidant micronutrients, and physical activity level of the patients over the study

**Variables**	**Propolis** **(N=23)**	**Placebo** **(N=21)**	***P***
Energy(kcal)			
Baseline	1766.52 ± 282.86	1706.05 ± 257.99	0.464^a^
End	1650.13 ± 266.694	1602.29 ± 296.04	0.962^c^
Mean difference (95%)	-116.39 (-191.03, -41.75)	-103.76 (-169.49, -38.03)	
P^*^	**0.004**	**0.004**	
Saturated fatty acids(gr)	
Baseline	16.69 ± 12	17.64 ± 10.83	0.769^b^
End	16.26 ± 8.19	16.77 ± 7.40	0.926^c^
Mean difference (95%)	-0.43 (-5.33, 4.47)	-0.87 (-4.98, 3.25)	
P^*^	0.903	0.794	
Polyunsaturated Fatty acid (gr)	
Baseline	15.06 ± 4.36	15.26 ± 4.44	0.879^a^
End	15.00 ± 4.45	12.61 ± 5.73	0.106^c^
Mean difference (95%)	-0.06 (-2.48, 2.36)	-2.65 (0.05, -5.26)	
P^*^	0.959	**0.046**	
Monounsaturated Fatty acid (gr)	
Baseline	13.21 ± 4.46	11.49 ± 5.73	0.272^a^
End	12.82 ± 7.28	12.34 ± 5.42	0.926^c^
Mean difference (95%)	-0.39 (-3.76, 2.99)	0.85 (-1.80, 3.50)	
P^*^	0.815	0.510	
Cholesterol (mg)			
Baseline	276.68 ± 158.96	274.02 ± 152.80	0.955^a^
End	287.18 ± 138.38	300.09 ± 130.50	0.640^c^
Mean difference (95%)	10.49 (-44.13, 65.11)	26.07 (-25.60, 77.74)	
P^*^	0.815	0.305	
Vitamin A (mg)			
Baseline	841.74 ± 861.93	703.68 ± 448.89	0.235^b^
End	603.99 ± 392.85	707.40 ± 406.38	0.556^d^
Mean difference (95%)	-237.75 (-626.44, 150.93)	3.71 (-306.98, 314.41)	
P^**^	0.855	0.741	
Vitamin C (mg)			
Baseline	87.15 ± 63.34	65.70 ± 42.20	0.254^b^
End	74.53 ± 46.87	57.73 ± 24.20	0.668^d^
Mean difference (95%)	-12.62 (-35.08, 9.84)	-7.96 (-30.05, -14.12)	
P^**^	0.201	0.876	
Vitamin E (mg)			
Baseline	7.51 ± 9.72	5.32 ± 6.49	0.391^b^
End	7.15 ± 8.04	4.68 ± 3.95	0.746^d^
Mean difference (95%)	-0.36 (-2.14, -1.41)	-0.64 (-2.17, 0.89)	
P^**^	0.394	0.808	
Selenium (mg)			
Baseline	0.09 ± 0.03	0.08 ± 0.02	0.238^a^
End	0.07 ± 0.03	0.08 ± 0.03	0.113^c^
Mean difference (95%)	-0.01 (-0.031, 0.004)	-0.01 (-0.016, 0.003)	
P^*^	0.116	0.190	
Zinc (mg)			
Baseline	7.9 ± 2.46	7.45 ± 2.09	0.879^b^
End	7.64 ± 2.6	8.21 ± 2	0.481^d^
Mean difference (95%)	-0.27 (-1.37, 0.83)	0.76 (-0.57, 2.1)	
P^**^	0.362	0.476	
Copper (mg)			
Baseline	1.1 ± 0.66	0.94 ± 0.23	0.488^b^
End	0.88 ± 0.22	0.84 ± 0.33	0.253^d^
Mean difference (95%)	-0.21 (-0.52, 0.10)	-0.10 (-0.23, 0.04)	
P^**^	0.191	0.164	
PA (METs)			
Baseline	444.70 ± 180.37	427.07 ± 173.98	0.744^a^
End	437.22 ± 200.28	422.71 ± 173.28	0.908^c^
Mean difference (95%)	-7.48 (-41.23, 26.57)	-4.36 (-35.91, 27.20)	
P^*^	0.653	0.776	

PA, physical activity; METs, metabolic equivalents (MET-min/week). Mean (SD) and mean difference (95% CI) are presented for data. *P* < 0.05 is defined as a significant level (Bold numbers). ^*^Paired samples t-test ^**^ Wilcoxon signed-rank test ^a^ Independent sample t-test. ^b^ Mann-Whitney test ^c^ ANCOVA test (adjusted for baseline values). ^d^ Quantile regression (adjusted for baseline values).

Table 3demonstrates serum levels of PAB as well as oxidative stress biomarkers at baseline and at the end of the study. Results showed that there was a significant reduction in PAB levels over the course of the trial in both groups. However, the inter-group difference was not significant at the endpoint. At the end of the study, the inter-group comparison showed a significant decrease in serum GPX level in the placebo group compared to the PRP group after adjusting for confounding variables based on models 1 (*P* = 0.027) and 2 (*P* = 0.028). No significant intra- or inter-group differences in other studied oxidative stress biomarkers, including MDA, SOD, and TAC, were found. Although the serum levels of ALT (*P* = 0.002), AST (*P* = 0.018),and GGT (*P* < 0.001) decreased significantly in the PRP group, between-group differences were not significant for these parameters (data are reported in our previously published article).^48^ However, the NAFLD fibrosis score improved significantly in the PRP group compared to the placebo group after adjusting for confounding factors (*P* = 0.021).

**Table 3:** Antioxidant and oxidative stress markers and body composition of the study participants throughout the study

Variables	**Propolis** **(N=23)**	**Placebo** **(N=21)**	***P***
PAB (HK)			
Baseline	96.63 ± 27.01	73.61 ± 24.45	23.02 (7.29, 38.74), 0.05^a^
End	75.29 ± 22	59.43 ± 17.07	-9.23 (-21.68, 3.21), 0.142^b^, 0.147^c^
Mean difference (95%)	-21.34 (-34.12, -8.52)	-14.18 (-23.85, -4.5)	
P^*^	**0.002**	**0.006**	
TAC (mmol/l)			
Baseline	1.51 ± 0.28	1.53 ± 0.35	0.02 (-018, 0.21), 0.868^a^
End	1.48 ± 0.32	1.49 ± 0.35	0.001(-0.149, 0.151), 0.993^b^, 0.995^c^
Mean difference (95%)	-0.038 (-0.16, 0.08)	-0.04 (-0.15, 0.07)	
P^*^	0.516	0.419	
MDA (nmol/ml)			
Baseline	1.88 ± 0.53	1.75 ± 0.48	0.02 (-0.35,0.39), 0.935^a^
End	1.83 ± 0.60	2.07 ± 0.79	-0.23 (-0.64, 0.19), 0.089^b^, 0.095^c^
Mean difference (95%)	-0.04 (-0.38, 0.29)	0.17 (-0.17, 0.52)	
P^*^	0.791	0.313	
GPX (U/gHb)			
Baseline	52.13 ± 14.43	53.99 ± 12.73	-1.86 (-10.17, 6.45), 0.654^a^
End	48.40 ± 17.12	39.61 ± 11.66	**-9.57 (-17.99, -1.15), 0.027** ^b^ **, 0.028** ^c^
Mean difference (95%)	-3.73 (-11.16, 3.71)	-14.38 (-20.76, -8.00)	
P^*^	0.310	**<0.001**	
SOD (U/gHb)			
Baseline	1278.19 ± 238.83	1398.54 ± 197.05	-91.40 ( -210.55, 27.76), 0.077^a^
End	1357.47 ± 202.48	1333.04 ± 147.83	-39.26 (-150.72, 72.19), 0.481^b^, 0.492^c^
Mean difference (95%)	79.28 (-31.84, 190.40)	-65.49 (-150.50, 19.51)	
P^*^	0.153	0.124	
BMI (kg/m^2^)			
Baseline	33.37 ± 4.82	33.00 ± 4.18	0.36 (-2.40, 3.12), 0.792^a^
End	32.03 ± 5.27	31.88 ± 4.48	0.24 (-0.40, 0.88), 0.460^b^, 0.503^c^
Mean difference (95%)	-1.34 (-1.88, -0.80)	-1.12 (-1.51, -0.74)	
P^*^	**<0.001**	**<0.001**	
FFM (kg)			
Baseline	62.54 ± 11	60.50 ± 9.45	2.04 (-4.23, 8.31), 0.514^a^
End	61.45 ± 11.66	58.79 ± 8.84	-0.64 (-2.01, 0.72), 0.346^b^, 0.329^c^
Mean difference (95%)	-1.09 (-2.10, -0.08)	-1.79 (-2.64, -0.78)	
P^*^	**0.036**	**0.001**	
FM (kg)			
Baseline	30.43 ± 9.31	27.96 ± 8.78	2.47 (-3.05, 7.99), 0.372^a^
End	29.02 ± 11.19	26.91 ± 9.54	0.55 (-1.76, 2.86), 0.633^b^, 0.696^c^
Mean difference (95%)	-1.41 (-3.55, 0.72)	-1.05 (-1.85, -0.25)	
P^*^	0.184	**0.012**	
FFM (%)			
Baseline	67.21 ± 7.03	68.17 ± 6.77	-0.96(-5.17, 3.25), 0.648^a^
End	68.84 ± 7.31	68.77 ± 7.09	-1.02 (-2.39, 0.35), 0.141^b^, 0.158^c^
Mean difference (95%)	1.63 (0.45, 2.80)	0.60 (-0.08, 1.28)	
P^*^	**0.009**	0.082	
FM (%)			
Baseline	32.50 ± 7.24	31.40 ± 6.84	1.09 (-3.21, 5.39), 0.611^a^
End	31.23 ± 7.60	31.00 ± 7.19	0.86 (-0.60, 2.37), 0.241^b^, 0.272^c^
Mean difference (95%)	-1.26 (-2.51, -0.01)	-0.40 (-1.11, 0.31)	
P^*^	**0.048**	0.256	
BW (%)			
Baseline	48.60 ± 5.37	43.50 ± 6.94	-0.76 (-4.00, 2.48), 0.505^a^
End	49.62 ± 5.61	42.15 ± 6.36	-0.91 (-1.99, 0.17), 0.097^b^, 0.102^c^
Mean difference (95%)	1.02 (0.08, 1.97)	-1.36 (-2.04, -0.67)	
P^*^	**0.035**	**0.001**	
NAFLD Fibrosis score			
Baseline	-2.21 ± 0.87	-2.21 ± 1.00	-0.003 (-0.57, 0.56), 0.990^a^
End	-2.47 ± 0.68	-1.97 ± 0.90	**0.50 (0.09, 0.91), 0.021** ^b^ **, 0.023** ^c^
Mean difference (95%)	-0.25 (-0.59, 0.09)	0.24 (-0.15, 0.63)	
P^*^	0.136	0.212	

MDA, malondialdehyde; TAC, Total antioxidant capacity; SOD, superoxide dismutase; GPX, Glutathione peroxidase; PAB, prooxidant-antioxidant balance; BMI, Body mass index; FFM, Fat free mass; FM, Fat mass; BW, Body water; NAFLD, Non-alcoholic fatty liver disease. Values are reported as mean ± standard deviation. *P* <0.05 is defined as a significant level (Bold numbers). * Paired samples t-test. ^a^ Independent sample t-test. ^c^ ANCOVA test, adjusted for baseline values (model 1). ^b^ ANCOVA test, adjusted for baseline values and energy changes (model 2).

 Although BMI significantly decreased in both groups, the between-group difference was not statistically significant after adjusting for baseline values and energy intake. We found a significant reduction in fat mass (FM) (%) and a significant increase in fat-free mass (FFM) (%) in the PRP group (*P* = 0.048 and *P* = 0.009, respectively). However, no significant between-group differences were observed for these parameters at the end of the study. Moreover, although there was a significant increase in total body water (%) in the PRP group (*P* = 0.035) and a significant reduction in the placebo group (*P* = 0.001), the between-group differences did not reach statistical significance, even after adjusting for the confounders.

## Discussion

 As far as we know, the present clinical trial is the first study to examine the effects of PRP supplementation along with a weight loss diet on oxidative stress biomarkers in obese patients with NAFLD, particularly using the PAB assay.

 In our study, an individualized weight-loss diet was administered to each patient and followed for 8 weeks. There were no significant differences between the groups in terms of dietary intake of energy and antioxidant micronutrients, as well as physical activity level, after 8 weeks of intervention (Table 2). As a result, these factors were not deemed confounding. However, we adjusted all study outcomes for baseline variables and changes in energy intake. Moreover, although both groups showed significant improvements in anthropometric measures and body composition, no inter-group differences were found for these parameters after adjusting for the confounders at the end of the study (Table 3), which is in line with the results of some previous studies. For example, Soleimani et al^42,53^ investigated the effects of 500 and 900 mg of PRP supplementation for 4 weeks in athletes and NAFLD patients. They reported that there was no significant effect on weight, FM, or FFM. Indeed, the results of a recent meta-analysis confirmed that PRP does not impact body weight or BMI.^54^ However, Koya-Miyata et al examined the effect of PRP in a mouse obesity model induced by a high-fat diet. They found that the administration of PRP (with a dose of 5 and 50 mg twice a day) compared to the control group for 10 days led to a decrease in body weight gain and weight of visceral adipose tissue by decreasing mRNA expression related to fatty acid biosynthesis, which includes sterol regulatory element binding protein, fatty acid synthase, and acetyl-CoA carboxylase in the PRP-treated mice.^55^ We failed to find improvements in anthropometric measures and body composition, possibly due to the relatively short duration of our study.

 Regarding oxidative stress biomarkers, results revealed that GPX change in the PRP group was less than in the placebo group (-3.73 U/gHb vs. -14.38 U/gHb), and the inter-group difference was found statistically significant after adjusting for both baseline values and change in energy intake (*P*= 0.027) (Table 3). In addition, serum MDA level, as a simple indicator of fat oxidation,^56^ decreased in the PRP group compared with an increase in the placebo group, while SOD increased in the PRP group compared with a decrease in the placebo group. However, both intra- and inter-group changes were not statistically significant. Serum PAB level showed a greater reduction in the PRP group than in the placebo group (-21.34 HK vs. -14.18 HK), but the difference was not significant (*P*= 0.142). In some animal studies, the positive effect of PRP on oxidative stress biomarkers was reported.^35,37,57^ Kismet et al showed that different doses (100 and 200 mg/kg) of PRP for two weeks in rats with NAFLD significantly improved MDA and GPX levels.^35^ Other studies investigating the effect of PRP supplementation (900 mg/d) in patients with T2DM for 18 weeks reported no significant effect on SOD, GPX, and MDA concentrations.^44,45^ However, PRP supplementation (1500 mg/d for 8 weeks) in T2DM patients had a significant increase in serum TAC, SOD, and GPX.^38^ PRP administration has also been reported to improve other oxidative stress biomarkers, such as catalase (CAT) and oxidized LDL, in T2DM patients.^43^ In the study by Darvishi et al, 250 mg PRP supplementation a day for a week before chemotherapy led to a significant improvement in oxidant/antioxidant balance compared to baseline.^47^ Indeed, the results of our recent meta-analysis of controlled clinical trials confirmed that PRP does not influence SOD and MDA but has a beneficial effect on GSH, GPX, and TAC levels.^58^ The antioxidant property of PRP is mainly related to the composition of flavonoids and polyphenols.^59^ It varies according to geographical location, source, and season of collection,^60,61^ which may explain the discrepancy between the results of different studies. Flavonoids inhibit radical species, protect antioxidant defense, and suppress reactive oxygen species (ROS) formation by inhibiting enzymes and chelating elements involved in the production of free radicals.^62^ Furthermore, PRP prevents oxidative stress by affecting the nuclear factor erythroid 2-related factor 2-antioxidant response element (Nrf2-ARE) pathway and increasing antioxidant response element (ARE) activation^63^ which is a main endogenous cellular defense mechanism against oxidative stress.^64^ Moreover, ethanolic extracts of Baccharis PRP, mainly containing artepillin C, camphoride, and chlorogenic acid, enhance nuclear translocation of Nrf2 and induce expression of thioredoxin reductase-1 (TRX-1), glutamate-cysteine ligase modifier subunit (GCLM), and heme oxygenase-1 (HO-1) in RAW 264.7 cells.^65^

 Although we observed significant reductions in serum liver enzymes, including ALT, AST, and GGT, between-group differences were not statistically significant. In addition, the AST/ALT ratio increased significantly in the placebo group, with no significant change in the PRP group. However, inter-group differences were not statistically significant (data are shown in our previously published article).^48^ Similarly, a previous study evaluating the efficacy of PRP (500 mg/d for 4 months) on hepatic steatosis and fibrosis in patients with NAFLD reported that PRP had no effect on serum levels of AST, ALT, GGT, and alkaline phosphatase in these patients.^53^ Nevertheless, some clinical trials with different designs have reported the beneficial effects of PRP supplementation on serum liver enzymes. For example, Silveira et al found that PRP administration (500 mg/d for 12 months) could prevent the increase of serum ALT in patients with chronic kidney disease.^66^ The discrepancy between the findings of studies may be due to differences in dosages of PRP, duration of the supplementation, and differences in clinical conditions. In the present study, we found that PRP supplementation led to a significant improvement in the NAFLD fibrosis score. Similarly, Kismet et al reported that PRP has positive effects on the histopathological and biochemical parameters of NAFLD, and these effects are related to the antioxidant and anti-inflammatory effects of PRP.^35^ Our finding was also in agreement with that of Soleimani et al, reporting the protective effects of PRP (50 mg twice daily for 4 months) on hepatic steatosis and fibrosis.^53^

 To the best of our knowledge, this trial appears to be the first study investigating PRP effects on oxidative stress biomarkers in patients with NAFLD. As an effective strategy for treating NAFLD, our participants received a CRD and were monitored every two weeks. However, the short duration of the study and the lack of a liver biopsy for NAFLD confirmation because of ethical problems are considered limitations of the present study.

## Conclusion

 Consuming Iranian PRP supplementation (1500 mg/d) for eight weeks could prevent the reduction of GPX levels compared to the control group. However, it could not affect other oxidative stress biomarkers, body composition, or dietary intakes of energy and antioxidant micronutrients. Further clinical trials with larger sample sizes and longer durations are recommended.

## Acknowledgments

 We thank the ‘Research Vice-Chancellor’ of Tabriz University of Medical Sciences, Tabriz, Iran for financial support. Also we sincerely thank the patients who cooperated in this study.

## Competing Interests

 The authors declare that they have no competing interests

## Ethical Approval

 All patients completed and signed the consent form after knowing the study objectives and protocol. This trial was approved by the Ethics Committee of the Research vice-chancellor (ethical code: TBZMED. REC.1399.942) as well as registered on the website www.irct.ir with the code (IRCT20100209003320N20).
